# Timing of vasopressin initiation and mortality in patients with septic shock: analysis of the MIMIC-III and MIMIC-IV databases

**DOI:** 10.1186/s12879-023-08147-6

**Published:** 2023-04-03

**Authors:** Jun Xu, Hongliu Cai, Xia Zheng

**Affiliations:** 1 Intensive Care Unit, The First Affiliated Hospital, Zhejiang University School of Medicine, 79 Qingchun Road, Hangzhou, 310003 Zhejiang Province P. R. China; 2 Key Laboratory of Clinical Evaluation Technology for Medical Device of Zhejiang Province, Hangzhou, Zhejiang Province P.R. China

**Keywords:** Vasopressin, Septic shock, Propensity score, MIMIC database, 28-day mortality

## Abstract

**Background:**

vasopressin is commonly used as a second-line vasopressor for patients with septic shock, but the optimal timing of initiation is uncertain. This study was designed to investigate when vasopressin initiation may be beneficial for 28-day mortality in septic shock patients.

**Methods:**

This was a retrospective observational cohort study from the MIMIC-III v1.4 and MIMIC-IV v2.0 databases. All adults diagnosed with septic shock according to Sepsis-3 criteria were included. Patients were stratified into two groups based on norepinephrine (NE) dose at the time of vasopressin initiation, defined as the low doses of NE group (NE<0.25 µg/kg/min) and the high doses of NE group (NE ≥ 0.25 µg/kg/min). The primary end‐point was 28‐day mortality after diagnosis of septic shock. The analysis involved propensity score matching (PSM), multivariable logistic regression, doubly robust estimation, the gradient boosted model, and an inverse probability‐weighting model.

**Results:**

A total of 1817 eligible patients were included in our original cohort (613 in the low doses of NE group and 1204 in the high doses of NE group). After 1:1 PSM, 535 patients from each group with no difference in disease severity were included in the analysis. The results showed that vasopressin initiation at low doses of NE was associated with reduced 28-day mortality (odds ratio [OR] 0.660, 95% confidence interval [CI] 0.518–0.840, p < 0.001). Compared with patients in the high doses of NE group, patients in the low doses of NE group received significantly shorter duration of NE, with less intravenous fluid volume on the first day after initiation of vasopressin, more urine on the second day, and longer mechanical ventilation-free days and CRRT-free days. Nevertheless, there were no significant differences in hemodynamic response to vasopressin, duration of vasopressin, and ICU or hospital length of stay.

**Conclusions:**

Among adults with septic shock, vasopressin initiation when low-dose NE was used was associated with an improvement in 28-day mortality.

## Background

Sepsis, defined as life-threatening organ dysfunction caused by a dysregulated host response to infection [1], is an enormous challenge in intensive care units (ICUs). It may lead to septic shock, multiorgan dysfunction or failure, and death, especially if not identified early and treated appropriately. Septic shock, characterized by severe hemodynamic failure, has a high mortality rate of over 40% [2]. Surviving Sepsis Campaign (SSC) Hour-1 sepsis bundle highlights lactate level, blood cultures, broad-spectrum antibiotics, rapid fluid resuscitation, and vasopressors support as a standard strategy for sepsis management [3]. To obtain a targeted mean arterial pressure (MAP) (≥ 65 mmHg) to ensure tissue perfusion, norepinephrine (NE) is currently recommended as the first-line vasoactive drug when fluid resuscitation fails. If MAP is still inadequate, the SSC guideline proposes to start a second-line vasopressor.

Vasopressin, an endogenous peptide hormone, is often regarded as a second-line agent to add to a low-to-moderate dose of NE for septic shock [3, 4]. Vasopressin may be useful owing to its norepinephrine-sparing effect. An epidemiology study about vasopressin for septic shock revealed that 17.2% of patients in United States hospitals received vasopressin, usually combined with catecholamines [5]. Initially, several small trials showed that patients with septic shock had a decrease in the required NE infusion when vasopressin was added but had no consistent effect on mortality [6–8]. Subsequently, both the Vasopressin and Septic Shock Trial (VASST) [9] and the Vasopressin versus Norepinephrine as Initial Therapy in Septic Shock (VANISH) [10] trial demonstrated that vasopressin therapy in septic shock did not affect 28-day or 90-day mortality although the confidence intervals were wide. A meta-analysis also showed that the use of vasopressin was not associated with decreased 28-day mortality [11], but may reduce the requirement for renal replacement therapy (RRT) [12]. However, in a subgroup analysis of the VASST trial, we found that vasopressin use seemed to benefit patients with less severe shock (26.5% vs. 35.7%, p = 0.05). A recent retrospective analysis suggested that the odds of in-hospital mortality increased by 20.7% for every increase of 10 µg/min NE-equivalent up to 60 µg/min at the time of vasopressin initiation in septic shock (adjusted OR 1.21, 95% CI 1.09–1.34) [13]. Therefore, the mixed results probably indicate that the key to improving outcomes in these patients is offering an earlier opportunity for vasopressin when the shock has not progressed to the point of irreversibility. More research is needed to discover whether early vasopressin initiation is associated with septic shock outcomes.

The SSC 2021 guidelines issue a weak recommendation for starting vasopressin initiation when the dose of NE is in the range of 0.25–0.5 µg/kg/min [3]. In our practice, the classic therapeutic strategy for septic shock is to apply NE and titrate to achieve a target MAP and then start a secondary agent if the MAP level is inadequate. Recently, Wieruszewski et al. pointed out that the classical stepwise strategy delayed obtaining adequate sustainable MAP and ultimately led to a poor prognosis [14]. A recent study showed that the use of NE dose in µg/min instead of µg/kg/min might reduce a delay of vasopressin initiation, particularly in obese patients [13]. High doses of NE at vasopressin initiation may be associated with an increased risk of mortality [15]. Thus, when to start vasopressin in septic shock is debatable and less clear.

We hypothesized that vasopressin initiation is associated with lower mortality when adults with septic shock are treated with low doses of NE. In response to this question, this study was conducted to evaluate the impact of vasopressin initiation on 28-day mortality in patients with septic shock from the Medical Information Mart in Intensive Care (MIMIC)-III v1.4 and MIMIC-IV v2.0 databases.

## Methods

### Database

This was a retrospective observational cohort study in which data was obtained from MIMIC-III v1.4 and MIMIC-IV v2.0 databases. MIMIC-III v1.4 is a famous single-center and freely accessible database, which includes over 40,000 critically ill patients at the Beth Israel Deaconess Medical Center (BIDMC) [16, 17]. MIMIC-IV v2.0 is the latest update to MIMIC-III, which adopts a modular approach to data organization and incorporates contemporary data [18]. We had completed the CITI Program course known as Human Research and Data or Specimens Only Research to apply for permission to access the database (Record ID: 48,615,099). The individual information of the patients included in this database was anonymous, and ethical review and informed consent were waived.

### Patients

**Fig. 1:**
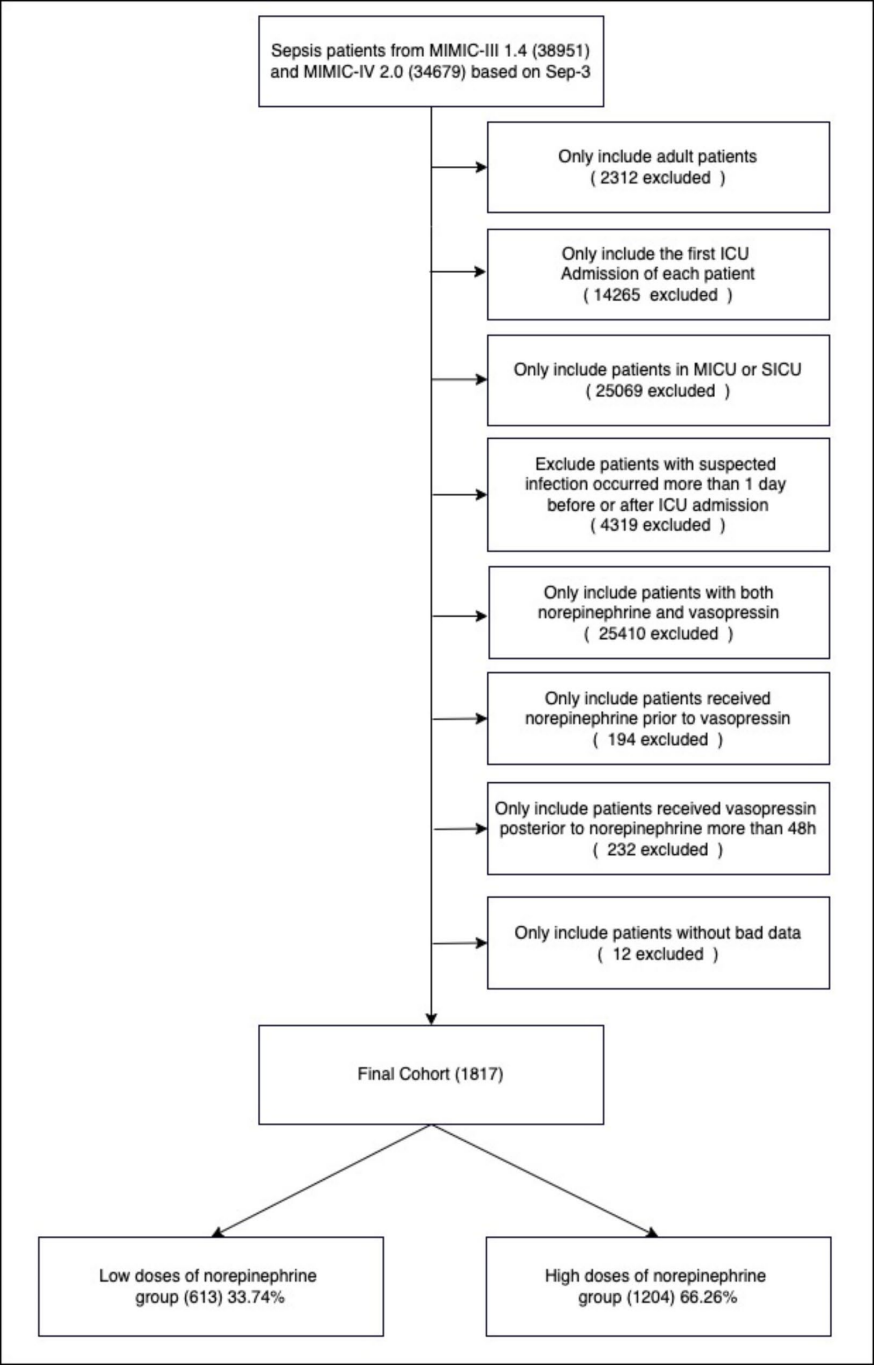
Study design flow chart. Illustration of exclusion and inclusion criteria as utilized to select the final cohort of 1817 patients

Our inclusion criteria were as follows: 1)adults (age ≥ 18 years old) were admitted to ICU with a diagnosis of sepsis based on Sepsis-3 criteria 1), which occurred less than one day before or after ICU admission, 2) patients came from the MICU or SICU and only the record of first ICU admission was used, 3) they had received both NE and vasopressin therapy, 4) NE initiation was prior to vasopressin and no more than 48 h. The missing > 10% of individual data were excluded. The mean imputation was used for continuous variables with missing values of < 10%. The patient inclusion flowchart is presented in detail in Fig. 1.

The SSC 2021 guidelines suggest adding vasopressin instead of escalating the dose of NE when it is in the range of 0.25–0.5 µg/kg/min [3]. Therefore, based on the NE dose at the time of vasopressin initiation, patients were classified into two groups: the low doses of NE group (NE<0.25 µg/kg/min) and the high doses of NE group (NE ≥ 0.25 µg/kg/min).

### Primary and secondary outcomes

The primary end-point was 28‐day mortality after diagnosis of septic shock. Secondary outcomes included the hemodynamic response to vasopressin, the duration of NE and vasopressin, the duration of mechanical ventilation-free days and CRRT-free days at day 28, intravenous fluid and urine on days 1, 2 after initiation of vasopressin, and the duration of ICU and hospital stays.

### Variables

The following variables were included in our study: age, gender, weight, ethnicity, service unit, comorbidities (diabetes mellitus, congestive heart failure, chronic obstructive pulmonary disease, chronic renal disease, liver disease, stroke, malignancy, and metastatic cancer), severity of illness scores at ICU admission (SOFA, LODS, and SAPS II scores), blood culture positivity, urine, the maximum serum lactate level between 6 h prior to shock onset and vasopressin initiation, and onset time of septic shock. Treatments (mechanical ventilation, CRRT, antibiotics, fluids administration, norepinephrine, vasopressin, and other vasoactive agents) at vasopressin initiation during the shock course were also required, as well as their start and end times.

The Sepsis-3 criteria for sepsis were extracted as suspected infection with associated organ dysfunction (SOFA ≥ 2) [1]. The onset time of septic shock was defined as the first time of NE initiation and a MAP of < 65 mm Hg was recorded. Fluid bolus volume was defined as any fluid bolus given from 6 h prior to shock onset through the time of vasopressin initiation. Hemodynamic response to vasopressin was defined as the achievement of both at least a 20% decrease from baseline in norepinephrine dose and MAP ≥ 65 mm Hg at 6 h after initiation of vasopressin [13]. When the patients were alive, the mechanical ventilation-free days and CRRT-free days were calculated as the number of days out of 28 days. If patients died within 28 days, the free days were defined zero.

### Statistical methods

To reduce the influence of potential confounders between the two groups, propensity score matching (PSM) analysis was conducted with the 1:1 optimal matching method and a caliper width of 0.02 by the “MatchIt” package in R software. And then, we examined the effects of vasopressin initiation in the two groups on the primary and secondary outcomes.

Another PSM analysis was conducted based on the gradient boosted model (GBM) with the “twang” package for the sensitivity analysis. The estimated propensity scores were calculated using a regression tree with all covariates in GBM. Subsequently, we built an inverse probabilities weighting (IPW) model for generating a weighted cohort with the estimated propensity scores as weights [19]. Logistic regression was then performed on the weighted cohort, adjusting for the potential confounders between the two groups in the propensity score model, thus called doubly robust analysis. The doubly robust analysis combines a multivariate regression model with a propensity score model to estimate the causal effect of an exposure on an outcome [20, 21]. In multivariate logistic regression analysis, variables with a p-value < 0.1 in the univariate analysis were considered candidate variables. All models were performed to assess the effect of early vasopressin initiation on the primary outcome. The odds ratio (OR) of 28-day mortality with a corresponding 95% confidence interval (CI) was calculated.

Continuous variables were expressed as mean ± standard, and categorical variables were expressed as proportions. We compared all covariates in the original and matched cohort. Statistical significance was tested with Student’s t-test for continuous variables and the χ2 test or Fisher’s exact test for categorical variables. Statistical significance was defined as a two-sided p-value of < 0.05. All statistical analyses were performed using R software (v4.2.1; The R Foundation for Statistical Computing).

### Sensitivity analysis

The sensitivity analysis was conducted to evaluate the robustness of the result of our study about the primary outcome. Four association inference models were also applied to understand how to affect our conclusion, which included a doubly robust model with unbalanced or all covariates, a propensity score-based IPW model, and a multivariable logistic regression model. These models’ calculated effect sizes and p values were reported and compared.

## Results

### Cohort characteristic

After reviewing 38,951 MIMIC-III and 34,679 adults with sepsis based on Sepsis-3, we identified 1817 patients in the final cohort who met the inclusion and exclusion criteria (Fig. 1). Based on the dose of NE at vasopressin initiation, 613 (33.74%) were assigned to the low doses of NE group and 1024 (66.26%) to the high doses of NE group. The baseline characteristics of the cohort are summarized in Table 1.

**Table 1:** Comparison of the basic demographics, comorbidities, and time of vasopressin initiation between the original cohort and the matched cohort

Variables	Original cohort				Matched cohort			Missing data (%)
	Low doses of NE group	High doses of NE group	p-value		Low doses of NE group	High doses of NE group	p-value	
n	613	1204			535	535		NA
**Characteristics at ICU admission**								
Age, year, mean ± SD	65.3 ± 15.9	65.5 ± 15.7	0.883		65.3 ± 16.0	65.9 ± 15.9	0.576	0.0
Male, n (%)	334 (54.5)	655 (54.4)	1.000		286 (53.5)	294 (55.0)	0.668	0.0
Service unit, n (%)			0.476				0.695	0.0
MICU	497 (81.1)	994 (82.6)			432 (80.7)	438 (81.9)		
SICU	116 (18.9)	210 (17.4)			103 (19.3)	97 (18.1)		
Weight, kg, mean ± SD	83.6 ± 24.5	82.8 ± 25.2	0.536		82.9 ± 23.9	83.6 ± 26.5	0.661	2.9
Race			0.974				0.966	0.0
White	379 (61.8)	755 (62.7)			329 (61.5)	338 (63.2)		
Black	60 (9.8)	108 (9.0)			53 (9.9)	50 (9.3)		
Hispanic	18 (2.9)	36 (3.0)			15 (2.8)	17 (3.2)		
Asian	19 (3.1)	41 (3.4)			17 (3.2)	16 (3.0)		
Other	137 (22.3)	264 (21.9)			121 (22.6)	114 (21.3)		
Comorbidities, n (%)								
Diabetes mellitus	189 (30.8)	352 (29.2)	0.516		164 (30.7)	165 (30.8)	1.000	0.0
Chronic heart failure	190 (31.0)	349 (29.0)	0.405		162 (30.3)	176 (32.9)	0.393	0.0
COPD	146 (23.8)	268 (22.3)	0.490		128 (23.9)	136 (25.4)	0.620	0.0
Chronic renal disease	126 (20.6)	271 (22.5)	0.372		118 (22.1)	107 (20.0)	0.453	0.0
Liver disease	161 (26.3)	353 (29.3)	0.190		143 (26.7)	131 (24.5)	0.441	0.0
Stroke	57 (9.3)	115 (9.6)	0.929		51 (9.5)	48 (9.0)	0.833	0.0
Malignancy	123 (20.1)	232 (19.3)	0.732		103 (19.3)	102 (19.1)	1.000	0.0
Metastatic cancer	54 (8.8)	115 (9.6)	0.667		47 (8.8)	54 (10.1)	0.530	0.0
Scoring system, mean ± SD								
SOFA	8.6 ± 4.5	8.9 ± 4.5	0.204		8.6 ± 4.5	8.5 ± 4.4	0.613	0.0
LODS	9.1 ± 3.4	10.4 ± 3.3	< 0.001		9.5 ± 3.4	9.4 ± 3.2	0.440	0.0
SAPS II	53.3 ± 15.3	59.4 ± 16.3	< 0.001		54.6 ± 15.3	54.5 ± 16.0	0.870	0.0
Blood culture positive, n (%)	171 (27.9)	302 (25.1)	0.217		141 (26.4)	136 (25.4)	0.780	0.0
Characteristics at vasopressin initiation								
Mechanical ventilation, n (%)	155 (25.3)	416 (34.6)	< 0.001		150 (28.0)	141 (26.4)	0.583	0.0
CRRT, n (%)	27 (4.4)	108 (9.0)	0.001		27 (5.0)	26 (4.9)	1.000	0.0
SOFA, mean ± SD	10.7 ± 4.2	11.2 ± 4.0	0.009		10.8 ± 4.2	10.7 ± 4.0	0.801	0.0
NE dose, µg/kg/min, mean ± SD	0.14 ± 0.07	0.43 ± 0.39	< 0.001		0.14 ± 0.07	0.41 ± 0.28	< 0.001	0.0
Maximum lactate, mmol/L, mean ± SD	5.5 ± 4.6	7.3 ± 5.3	< 0.001		5.8 ± 4.8	6.0 ± 4.6	0.465	3.3
Time from shock onset to vasopressin, hour, mean ± SD	11.1 ± 12.1	7.8 ± 10.0	< 0.001		10.3 ± 11.7	9.9 ± 11.6	0.579	0.0
Medication Data during Shock Course								
Antibiotics, n (%)	585 (95.4)	1154 (95.8)	0.772		508 (95.0)	510 (95.3)	0.887	0.0
IV hydrocortisone, n (%)	138 (22.5)	389 (32.3)	< 0.001		129 (24.1)	135 (25.2)	0.723	0.0
Fluid bolus volume, mL, mean ± SD	3446 ± 3796	3156 ± 3635	0.114		3386 ± 3812	3328 ± 3580	0.797	0.0
Dopamine, n (%)	49 (8.0)	157(13.0)	0.002		47 (8.8)	54 (10.1)	0.530	0.0
Dobutamine, n (%)	40 (6.5)	70 (5.8)	0.619		34 (6.4)	38 (7.1)	0.714	0.0
Epinephrine, n (%)	46 (7.5)	220 (18.3)	< 0.001		46 (8.6)	46 (8.6)	1.000	0.0

Abbreviations: ICU = intensive care unit; SD = standard deviation; MICU = medical intensive care unit; SICU = surgery intensive care unit; COPD = chronic obstructive pulmonary disease; SOFA = sequential organ failure assessment; LODS = logistic organ dysfunction system; SAPS II = simplified acute physiology score II; CRRT = continuous renal replacement therapy; NE = norepinephrine

In the original cohort, there were significant differences between the two groups in LODS and SAPS II scores at ICU admission; the proportion of patients receiving mechanical ventilation and CRRT; SOFA score and maximum lactate levels at the time of vasopressin initiation; time from shock onset to vasopressin; and medication during shock course, including hydrocortisone, dopamine, dobutamine, and epinephrine.

**Fig. 2:**
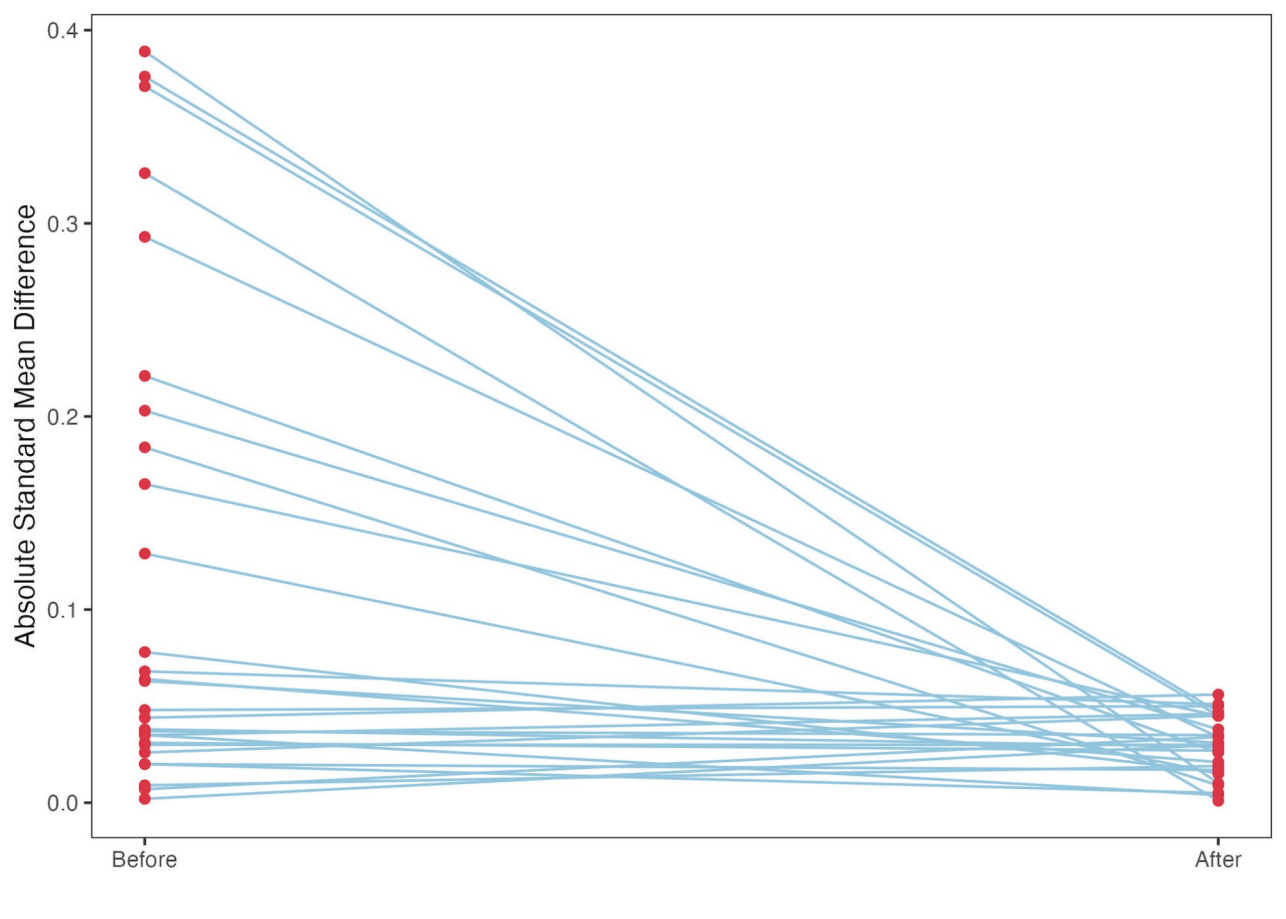
Absolute standard difference of covariates between groups for the original cohort and the matched cohort

After 1:1 PSM, 535 patients from each group were included in the analysis. All baseline characteristics were well-balanced after PSM, and there was no significant difference in disease severity between the two groups before the time of vasopressin initiation. We compared the absolute standard difference between the original and the matched cohorts (Fig. 2).

### Primary outcome and sensitivity analysis

In the matched cohort, the mean NE dose was 0.14 ± 0.07 µg/kg/min in the low doses of NE group and 0.41 ± 0.28 µg/kg/min in the high doses of NE group at the time of vasopressin initiation. We found that the 28-day mortality rates for the low and high doses of NE groups were 49.2% vs. 59.4% (OR 0.660, 95% CI 0.518–0.840, p < 0.001). For the sensitivity analysis, the other four association inference models reached the same conclusion: When adults with septic shock were treated with low-dose NE, initiation of vasopressin therapy was associated with a reduction in 28-day mortality. Primary outcome analysis with five different models is summarized in Table 2.

**Table 2:** Primary outcome analysis with five different models

Method	OR	CI		*P*-value
		2.5%	97.5%	
Propensity score matching	0.660	0.518	0.840	<0.001
Propensity score IPW	0.622	0.539	0.718	<0.001
Doubly robust with unbalanced covariates	0.637	0.504	0.806	<0.001
Doubly robust with all covariates	0.635	0.499	0.809	<0.001
Multivariable logistic regression	0.604	0.484	0.753	<0.001

OR = odds ratio; CI = confidence interval; IPW = inverse probability weighting

**Fig. 3:**
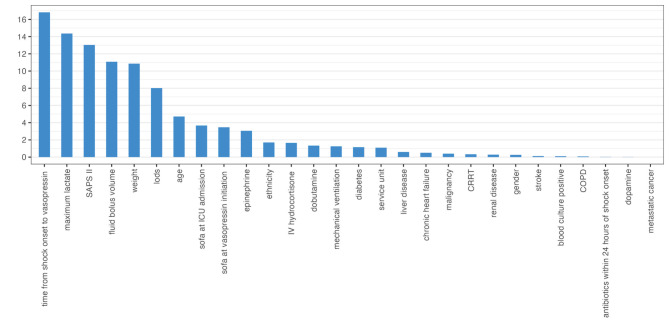
Relative contributions of individual covariates to the final propensity score

In addition, we listed the contribution of each covariate to the propensity score in GBM (Fig. 3). The top 5 covariates include time from shock onset to vasopressin, maximum lactate levels at the time of vasopressin initiation, SAPS II score at ICU admission, fluid bolus volume, and weight.

### Secondary outcomes with PSM

In the matched cohort, there was no significant difference in hemodynamic response to vasopressin (61.1% in the low doses of NE group vs. 60.0% in the high doses of NE group; P = 0.754). Duration of vasopressin and ICU or hospital length of stay also did not significantly differ between the groups. Patients in the low doses of NE group received significantly shorter duration of NE, less intravenous fluid volume on day 1 and more urine on day 2 after initiation of vasopressin, and longer duration of mechanical ventilation-free days and CRRT-free days than patients in the high doses of NE group. The detailed results are summarized in Table 3.

**Table 3:** Primary and secondary outcomes analysis between the original cohort and the matched cohort

Outcomes	Original cohort			Matched cohort			Missing data (%)
	Low doses of NE group	High doses of NE group	p-value	Low doses of NE group	High doses of NE group	p-value	
n	613	1204		535	535		NA
**Primary outcome**							
28-day mortality, n (%)	286 (46.7)	781 (64.9)	< 0.001	263 (49.2)	318 (59.4)	< 0.001	0.0
**Secondary outcomes**							
Hemodynamic response to vasopressin, n (%)	383 (62.5)	628 (52.5)	< 0.001	327 (61.1)	321 (60.0)	0.754	0.0
Norepinephrine duration, hours, mean ± SD	63.5 ± 73.3	70.9 ± 91.3	0.082	63.9 ± 71.3	76.0 ± 89.2	0.014	0.0
Vasopressin duration, hours, mean ± SD	49.7 ± 62.1	48.6 ± 66.7	0.747	50.8 ± 63.9	51.6 ± 71.2	0.859	0.0
Mechanical ventilation-free days, mean ± SD	12.2 ± 13.1	7.4 ± 11.3	< 0.001	11.7 ± 13.1	8.8 ± 12.1	< 0.001	0.0
CRRT-free days, mean ± SD	14.6 ± 14.4	9.2 ± 13.2	< 0.001	13.9 ± 14.4	11.0 ± 13.9	0.001	0.0
IV fluid day 1 (mL)	4578 ± 3287	6046 ± 4396	< 0.001	4739 ± 3372	5352 ± 3892	0.006	0.0
IV fluid day 2 (mL)	2453 ± 2385	2616 ± 2933	0.242	2479 ± 2431	2532 ± 2770	0.741	2.1%
Urine day 1 (mL)	1049 ± 1201	777 ± 1167	< 0.001	999 ± 1214	936 ± 1314	0.415	0.0
Urine day 2 (mL)	1158 ± 1225	797 ± 1076	< 0.001	1087 ± 1188	899 ± 1130	0.012	13.1%
Duration of ICU stays, days, mean ± SD	8.2 ± 8.0	7.3 ± 8.9	0.029	8.1 ± 8.0	7.7 ± 8.6	0.455	0.0
Duration of hospital stays, days, mean ± SD	14.9 ± 14.9	12.3 ± 15.4	0.001	14.7 ± 14.7	13.5 ± 14.9	0.186	0.0

Abbreviations: ICU = intensive care unit; SD = standard deviation; CRRT = continuous renal replacement therapy; IV = intravenous; NE = norepinephrine

## Discussion

Septic shock, a subset of sepsis, is clinically identified by persisting hypotension requiring vasopressors and serum lactate levels greater than 2 mmol/L despite adequate fluid resuscitation [1]. In the 2021 SSC guidelines, NE is strongly recommended as the first-line vasopressor over other vasopressors for septic shock [3], with vague guidance on second-line selection and timing. This is a propensity-matched retrospective observational cohort study from MIMIC-III and MIMIC-IV databases. We evaluated the effect of vasopressin initiation on 28‐day mortality in patients with septic shock. We found a significantly lower 28‐day mortality among patients who initiated vasopressin when they were on low doses of NE after adjustment for confounding (OR 0.660, 95% CI 0.518–0.840, p < 0.001). The other four estimation models validated our main result and led to the same conclusion. Thus, our study proves that the optimal timing of vasopressin initiated can be earlier than recommended by guidelines, which provides a rationale for prospective studies.

Our main finding is consistent with several previous studies. In the VASST trial, there was no significant difference in mortality rates in the overall septic shock population. In contrast, patients with less severe shock receiving NE < 15 µg/min was associated with a lower mortality rate (28-day mortality risk ratio [RR] 0.74, 95% CI 0.55–1.01, P = 0.05; 90-day mortality RR 0.78, 95% CI 0.61–0.99, P = 0.04) [9]. A retrospective cohort study of 96 patients with septic shock showed that early intervention with vasopressin (within 4 h of shock onset) might achieve MAP targets sooner and resolve organ dysfunction at 72 h more effectively [22]. Subsequently, a single-center, prospective, open-label trial conducted by the same group demonstrated the same results [23]. In a large retrospective observational study, Sacha et al. found that each 10 µg/min increase in NE-equivalent dose up to 60 µg/min at the time of vasopressin initiation was associated with 20.7% higher in-hospital mortality [13], indicating patients could benefit from early vasopressin initiation. In addition, compared with catecholamines alone, the addition of vasopressin to catecholamine vasopressors was associated with a lower risk of atrial fibrillation [24].

However, a systematic review and meta-analysis showed that the administration of vasopressin was not associated with reduced 28-day or 30-day mortality among patients with septic shock. At the same time, an increased incidence of digital ischemia should be noted [25]. Another review also failed to show that short-term mortality decreased when vasopressin was initiated early within 6 h of septic shock onset, except for reducing the use of RRT [12]. But Young et al. [26] considered that it is inappropriate to conclude simply that vasopressin therapy does not affect mortality in septic shock because most ICU interventions have little plausible impact on mortality in a heterogeneous population. Sepsis is a heterogeneous population with different clinical phenotypes [27]. So, actual mega trials are needed if we want to understand how vasopressin affects survival.

To test several hypotheses for accounting for the mortality benefit, we compared several variables between the two groups. In matched cohort, we found that vasopressin initiation in the low doses of NE group could decrease the duration of NE, prolong the duration of mechanical ventilation-free days or CRRT-free days, and more urine on day 2 after initiation of vasopressin. Although no difference in kidney failure-free days was found in the VANISH trial, the 95% CI of the difference between groups has an upper limit of 5 days in favor of vasopressin [10]. Due to the distribution of V1a receptors in the kidney, vasopressin may maintain renal perfusion better than NE [6], which may explain our findings. In addition, more IV fluids were administered to the high doses of NE group on day 1 after vasopressin initiation. Fluid overload was associated with harm in the prospective FINNAKI study [25]. Considering the factors displayed in Fig. 3, clinicians may particularly want to consider early vasopressin initiation in patients with a high lactate, high SAPA II score, and massive fluid resuscitation. More research is needed to determine whether the 28-day mortality improvements are due to the differences in fluid admission, urine, duration of NE, mechanical ventilation-free days, and CRRT-free days.

There are other opinions on the optimal timing of vasopressin use. Wieruszewski et al. recently suggested using an early, multimodal balanced vasopressor strategy instead of a stepwise escalation of vasopressors, also named broad-spectrum vasopressors [14]. This is analogous to broad-spectrum and early antimicrobials in suspected and confirmed sepsis. Some studies pointed out that whether vasopressin was used or not could be based on the hemodynamic response after 1 U bolus vasopressin is administered [28] or a machine learning prediction model [29]. On the contrary, Jakowenko et al. found that early vasopressin initiation was not associated with responsiveness at 4 h post-catecholamine initiation, suggesting that it may be reasonable to implement the 2021 SSC guidelines [30]. An observational cohort study revealed that the vasopressin response appeared to be impaired in the setting of severe acidemia [31]. Surprisingly, the severity of illness defined by APACHE II and SOFA scores, corticosteroid use, and catecholamine dose were not associated with hemodynamic response [32]. Our study demonstrated that high doses of NE at vasopressin initiation in septic shock leaded to excessive catecholamine exposure, more fluid overload, prolonged recovery of kidney function, and poor outcome.

Our study has several limitations. First, although the MIMIC-III and MIMIC-IV databases included comprehensive and high-quality data, this was a single-center study, and caution should be exercised when applying results in other regions. Second, as a retrospective study, it was impossible to adjust for all confounders because of the potential issues of residual confounding. Although we used a PSM approach, it remains possible that the high doses of NE group had a higher mortality rate because of the severity of the disease. Third, the outcomes were not adjusted by year, which was a limitation of the analysis because treatment strategies for septic shock changed over time. Some results will require prospective randomized trials for confirmation.

## Conclusions

Among adults with septic shock, vasopressin initiation when low-dose NE was used was associated with an improvement in 28-day mortality. Further large-scale randomized controlled trials are needed to confirm this conclusion.

## Data Availability

The datasets are available from the corresponding author on reasonable request. The details of the data screening codes for our analyses, which were provided by the authors of the MIMIC III and IV database, can be found at GitHub (https://github.com/MIT-LCP/mimic-code).

## List of Abbreviations

MIMIC: Medical Information Mart in Intensive Care
NE: Norepinephrine
PSM: Propensity score matching
OR: Odds ratio
CI: Confidence interval
CRRT: Continuous renal replacement therapy
ICU: Intensive care unit
SSC: Surviving sepsis campaign
MAP: Mean arterial pressure
VASST: Vasopressin and Septic Shock Trial
VANISH: Vasopressin versus Norepinephrine as Initial Therapy in Septic Shock
BIDMC: Beth Israel Deaconess Medical Center
CITI: Collaborative Institutional Training Initiative
MIT: Massachusetts Institute of Technology
MICU: Medical intensive care unit
SICU: Surgery intensive care unit
SOFA: Sequential organ failure assessment
LODS: Logistic organ dysfunction system
SAPS II: Simplified acute physiology score II
GBM: Gradient boosted model
IPW: Inverse probabilities weighting
RR: Risk ratio
IV: Intravenous
APACHE II: Acute Physiology and Chronic Health Evaluation II

## References

[1] Singer M, Deutschman CS, Seymour CW, Shankar-Hari M, Annane D, Bauer M (2016). The Third International Consensus Definitions for Sepsis and septic shock (Sepsis-3). JAMA.

[2] Bauer M, Gerlach H, Vogelmann T, Preissing F, Stiefel J, Adam D (2020). Mortality in sepsis and septic shock in Europe, North America and Australia between 2009 and 2019— results from a systematic review and meta-analysis. Crit Care.

[3] Evans L, Rhodes A, Alhazzani W, Antonelli M, Coopersmith CM, French C (2021). Surviving sepsis campaign: international guidelines for management of sepsis and septic shock 2021. Intensive Care Med.

[4] Vincent J-L, Post EH (2016). Vasopressin: a first-line agent for septic shock?. Nat Rev Nephrol.

[5] Vail EA, Gershengorn HB, Hua M, Walkey AJ, Wunsch H. Epidemiology of Vasopressin Use for Adults with Septic Shock.Annals ATS. 2016;:AnnalsATS.201604-259OC. 10.1513/AnnalsATS.201604-259OC PMC5122493 27404213

[6] Landry DW, Levin HR, Gallant EM, Seo S, D’Alessandro D, Oz MC (1997). Vasopressin pressor hypersensitivity in vasodilatory septic shock. Crit Care Med.

[7] Patel BM, Walley KR (2002). Beneficial Effects of short-term vasopressin infusion during severe septic shock. Anesthesiology.

[8] Xiao X, Zhang J, Wang Y, Zhou J, Zhu Y, Jiang D (2016). Effects of terlipressin on patients with sepsis via improving tissue blood flow. J Surg Res.

[9] Russell JA, Hébert PC, Granton JT, Ayers D. Vasopressin versus Norepinephrine Infusion in Patients with Septic Shock.n engl j med. 2008;:11. 10.1056/NEJMoa067373 18305265

[10] Gordon AC, Mason AJ, Thirunavukkarasu N, Perkins GD, Cecconi M, Cepkova M (2016). Effect of early vasopressin vs norepinephrine on kidney failure in patients with septic shock: the VANISH Randomized Clinical Trial. JAMA.

[11] Nagendran M, Russell JA, Walley KR, Brett SJ, Perkins GD, Hajjar L (2019). Vasopressin in septic shock: an individual patient data meta-analysis of randomised controlled trials. Intensive Care Med.

[12] Huang H. The effect of early vasopressin use on patients with septic shock: A systematic review and meta-analysis.American Journal of Emergency Medicine. 2021;:6. 10.1016/j.ajem.2021.05.007 33975132

[13] Sacha GL, Lam SW, Wang L, Duggal A, Reddy AJ, Bauer SR. Association of Catecholamine Dose, Lactate, and Shock Duration at Vasopressin Initiation With Mortality in Patients With Septic Shock.Critical Care Medicine. 2021;Publish Ahead of Print. 10.1097/CCM.0000000000005317 34582425

[14] Wieruszewski PM, Khanna AK (2022). Vasopressor choice and timing in vasodilatory shock. Crit Care.

[15] Guerci P, Belveyre T, Mongardon N, Novy E (2022). When to start vasopressin in septic shock: the strategy we propose. Crit Care.

[16] Johnson AEW, Pollard TJ, Shen L, Lehman LH, Feng M, Ghassemi M (2016). MIMIC-III, a freely accessible critical care database. Sci Data.

[17] Johnson A, Pollard T, Mark R. MIMIC-III Clinical Database CareVue subset (version 1.4). PhysioNet. 2022.

[18] Johnson A, Bulgarelli L, Pollard T, Horng S, Celi LA, Mark R. MIMIC-IV (version 2.0). PhysioNet. 2022.

[19] Cole SR, Hernan MA (2008). Constructing inverse probability weights for marginal structural models. Am J Epidemiol.

[20] McCaffrey DF, Griffin BA, Almirall D, Slaughter ME, Ramchand R, Burgette LF (2013). A tutorial on propensity score estimation for multiple treatments using generalized boosted models. Statist Med.

[21] Funk MJ, Westreich D, Wiesen C, Stürmer T, Brookhart MA, Davidian M (2011). Doubly robust estimation of Causal Effects. Am J Epidemiol.

[22] Hammond DA, Cullen J, Painter JT, McCain K, Clem OA, Brotherton AL (2019). Efficacy and safety of the early addition of Vasopressin to Norepinephrine in Septic Shock. J Intensive Care Med.

[23] Hammond DA, Ficek OA, Painter JT, McCain K, Cullen J, Brotherton AL (2018). Prospective open-label trial of early concomitant vasopressin and norepinephrine therapy versus initial norepinephrine monotherapy in septic shock. Pharmacotherapy.

[24] McIntyre WF, Um KJ, Alhazzani W, Lengyel AP, Hajjar L, Gordon AC (2018). Association of Vasopressin Plus Catecholamine Vasopressors vs catecholamines alone with Atrial Fibrillation in patients with distributive shock: a systematic review and Meta-analysis. JAMA.

[25] Yao R, Xia D, Wang L, Wu G, Zhu Y, Zhao H (2020). Clinical efficiency of Vasopressin or its analogs in comparison with catecholamines alone on patients with septic shock: a systematic review and Meta-analysis. Front Pharmacol.

[26] Young PJ, Delaney A, Venkatesh B (2019). Vasopressin in septic shock: what we know and where to next?. Intensive Care Med.

[27] Seymour CW, Kennedy JN, Wang S, Chang C-CH, Elliott CF, Xu Z (2019). Derivation, validation, and potential treatment implications of Novel Clinical Phenotypes for Sepsis. JAMA.

[28] Nakamura K, Nakano H, Naraba H, Mochizuki M, Takahashi Y, Sonoo T (2021). Vasopressin loading for refractory septic shock: a preliminary analysis of a Case Series. Front Med.

[29] Scheibner A, Betthauser KD, Bewley AF, Juang P, Lizza B, Micek S et al. Machine learning to predict vasopressin responsiveness in patients with septic shock.Pharmacotherapy. 2022;:phar.2683. 10.1002/phar.2683 35426141

[30] Jakowenko ND, Murata J, Kopp BJ, Erstad BL. Influence of Timing and Catecholamine Requirements on Vasopressin Responsiveness in Critically ill Patients with Septic Shock.J Intensive Care Med. 2022;:088506662210818. 10.1177/08850666221081836 35195486

[31] Bauer SR, Sacha GL, Siuba MT, Lam SW, Reddy AJ, Duggal A (2022). Association of arterial pH with hemodynamic response to Vasopressin in patients with septic shock: an Observational Cohort Study. Crit Care Explorations.

[32] Sacha GL, Lam SW, Duggal A, Torbic H, Bass SN, Welch SC (2018). Predictors of response to fixed-dose vasopressin in adult patients with septic shock. Ann Intensive Care.

